# Structural, Thermal and Pasting Properties of Heat-Treated Lotus Seed Starch–Protein Mixtures

**DOI:** 10.3390/foods11192933

**Published:** 2022-09-20

**Authors:** Sidi Liu, Wenyu Chen, Changyu Zhang, Tong Wu, Baodong Zheng, Zebin Guo

**Affiliations:** 1 College of Food Science, Fujian Agriculture and Forestry University, Fuzhou 350002, China; 2 Fujian Provincial Key Laboratory of Quality Science and Processing Technology in Special Starch, Fujian Agriculture and Forestry University, Fuzhou 350002, China

**Keywords:** lotus seed starch, lotus seed protein, starch–protein mixture, structural properties, thermal properties

## Abstract

The interactions between starch and protein, the essential components of lotus seed, strongly influence the quality of lotus seed processing by-products. This study investigated the effects of lotus seed starch–protein (LS-LP) interactions on the structural, thermal and gelatinization properties of LS-LP mixtures, using LS/LP ratios of 6:1, 6:2, 6:3, 6:4, 6:5, or 1:1, after heat treatment (95 °C, 30 min). Fourier transform infrared peaks at 1540 cm^−1^ and 3000–3600 cm^−1^ revealed the major interactions (electrostatic and hydrogen bonding) between LS and LP. The UV–visible absorption intensities (200–240 nm) of LS-LP mixtures increased with increased protein content. X-ray diffraction and electron microscopy revealed that LS-LP consists of crystalline starch granules encapsulated by protein aggregates. Increasing the addition of protein to the mixtures restricted the swelling of the starch granules, based on their solubility, swelling properties and thermal properties. Viscometric analysis indicated that the formation of LS-LP mixtures improved structural and storage stability. These findings provide a practicable way to control the thermal and gelatinization properties of lotus seed starch–protein mixtures, by changing the proportions of the two components, and provide a theoretical basis for developing novel and functional lotus-seed-based foods.

## 1. Introduction

Starch and protein are the main components of many staple foods, have beneficial biological activities, and valuable functional properties, which provide nutritional and health benefits, and have a strong influence on the texture and taste of food. Therefore, the interactions between them have attracted extensive research attention in recent years [1]. Proteins contain many hydrophilic groups, such as amide, hydroxyl, carboxyl and thiol, which mediate the interactions between starch and protein [2,3]. There is extensive evidence that starch–protein mixtures are formed through electrostatic and van der Waals interactions, hydrogen bonding, and hydrophobic interactions [4], for example, electrostatic interactions and hydrogen bonding were the main bonding interactions in a potato starch–protein mixture [5]. The dominant intermolecular binding interactions in a particular starch–protein mixture depend on the starch/protein ratio, the molecular structures of the starch and protein and the processing conditions used to form the mixture [2].

The physicochemical properties of the starch and protein components change after mixture formation and these changes can include rheological properties [6], gelatinization properties [7], retrogradation properties [8], solubility [9] and gelling properties [10], all of which can strongly influence food quality and consumer acceptance. Many high-starch foods contain a large proportion of protein, so starch and protein can easily interact and form mixtures during cooking, or industrial food processing [11]. Therefore, many previous studies used extrusion cooking [12,13], or heat treatment [2,14,15,16] to simulate the formation of starch–protein mixtures during food production and processing. Moreover, this physical heating modification method is helpful to improve the surface activity of starch and thus to improve the possibility of interaction between starch and protein [17]. Heat treatment is generally more advantageous for research purposes because it is easy to control, requires only simple apparatus and is very widely used, both in domestic cooking and industrial production of processed foods. However, there is limited information on starch–protein interactions during thermal processing and their effects on the molecular structures, inter- and intra-molecular interactions and the thermal properties of starch [18]. Monitoring changes in thermal properties resulting from thermal processing of starch-based products is an excellent way to investigate intermolecular interactions in starch–protein mixtures. Previous reports on the effects of additional protein on thermal properties indicate that these studies have great potential, but extensive research remains to gain a full understanding. Therefore, an in-depth study of the interactions between starch and protein would be helpful to improve the theoretical understanding of these interactions, as well as the stability, texture and quality of starch-based foods [14].

Lotus seeds, from the fruit of Nelumbo nucifera Gaertn., are a good source of carbohydrates, alkaloids, proteins and amino acids, and have been made into various commercially-available food products, including instant powder, pastries, canned foods, juice and beverages [19]. Starch is a naturally rich polymer produced by several plants including lotus seeds, containing about 50% *w/w* starch (dry basis), which is mostly amylose (40% *w/w*) [20,21]. In addition to starch as the major component, lotus seeds contain about 20% *w/w* protein with a high biological value (BV = 90.06) [22]. Moreover, lotus seed protein is free of common allergens, which increases its potential applications in novel food products and widens the potential consumer base [23]. We previously reported the structure, physicochemical properties and formation mechanism of lotus seed starch and its starch-lipid and starch-polyphenol mixtures, under different treatment conditions [24,25]. 

This study involved the preparation of lotus seed starch–protein mixtures, using heat treatment to gelatinize the starch and disrupt its ordered structure, as well as denaturing the protein, facilitating the formation of stable mixtures with novel structures and physicochemical properties during cooling. The objective was to elucidate the formation mechanism of the mixtures by structural analysis at different scales (particle structure, aggregate structure, short-range structure), and determination of thermal and gelatinization properties. This information should increase the theoretical understanding of the formation and structure of starch–protein mixtures and facilitate the development of extensively-processed lotus seed products with specific characteristics.

## 2. Materials and Methods

### 2.1. Materials

Fresh lotus seeds were from Green Field Food Co. Ltd. (Fujian, China) and all other chemicals were of analytical grade, from Sinopharm Chemical Regent Co. Ltd. (Shanghai, China).

#### 2.1.1. Extraction of Lotus Seed Starch (LS)

Lotus seed starch was isolated as described previously [26], with minor modifications. The fresh lotus seeds were soaked in distilled water (30 min), pulverized in a grinder (Changzhou Xiangtian Experimentation Instrument Plant Co. Ltd., Changzhou, China), filtered through a 100-mesh sieve and the resulting suspension was allowed to stand at ambient temperature for 6 h. The precipitate which formed was washed three times with distilled water and three times with 95% alcohol, then dried in an oven (Shanghai Yiheng Technology Co. Ltd., Shanghai, China) overnight at 45 °C, pulverized, and passed through a 100-mesh sieve to obtain lotus seed starch powder. 

#### 2.1.2. Extraction of Lotus Seed Protein (LP)

Lotus seed protein was isolated as described previously, with minor modifications [27]. The dried lotus seeds were pulverized in a grinder, as above, then the resulting powder (500 g) was added to distilled water (5 L), adjusted to pH 11.0 with 0.5 mol L^−1^ NaOH, stirred for 3 h at room temperature and stored for 24 h at 4 °C. The supernatant was collected by centrifugation at 4500× *g* for 10 min, the pH adjusted to 4.8 by adding 0.1 mol L^−1^ HCl solution, the solution stirred for 3 h at room temperature and stored for 24 h at 4 °C. The precipitated protein was collected by centrifugation at 4500× *g* for 10 min, redissolved in deionized water (1:10, *w/v*) at neutral pH and lyophilized in a freeze dryer (FDU-1200, Tokyo Rikakikai Co., Ltd., Tokyo, Japan). Finally, lotus seed protein powders were obtained.

### 2.2. Preparation of Lotus Seed Starch–Protein Mixtures (LS-LP)

LS was dispersed in distilled water (6%, *w/v*) and then mixed with LP solutions of different concentrations (1, 2, 3, 4, 5, or 6% *w/v*). The pH of the mixtures was maintained at 7.4 by the addition of phosphate buffer and dispersed evenly. The mixtures were then cooked in a water bath at 95 °C with stirring for 30 min, cooled, stored for 48 h at 4 °C, freeze-dried, ground and passed through a 100-mesh sieve. The resulting LS-LP powders were stored in a desiccator until needed.

### 2.3. Fourier Transform Infrared (FTIR) Spectroscopy

The FTIR spectra were recorded on an FTIR spectrometer (Bruker, Karlsruhe, Germany) as described previously [28]. The samples (10 mg) and KBr (500 mg) were dried for 12 h at 105 °C, thoroughly mixed and formed into thin disks for spectrometry. The wavenumber range was 4000 to 400 cm^−1^, with a resolution of 4 cm^−1^.

### 2.4. UV–Visible Absorption Spectra

Spectra were recorded for standard barium sulfate sample cell on a UV-3600Plus (Shimadzu, Kyoto, Japan) over a wavelength range of 200–400 nm and a 1.0 cm path length as a baseline. Then the standard barium sulfate sample cell was taken out from the integrating sphere, and replaced with the sample cell pressed with all samples, as described previously [29].

### 2.5. X-ray Diffraction (XRD)

XRD analysis of the starch granules was performed with an X-ray diffractometer (Rigaku Corporation, Tokyo, Japan), as described previously with some modifications [30]. The scan range was 5° to 35° (2θ), 40 kV, 200 mA and Cu-Kα radiation. The relative crystallinity was calculated as the area ratio of the crystalline peak over the total area by using PeakFit 4.0 software (Beijing, China).

### 2.6. Scanning Electron Microscopy (SEM)

The surface morphology of granular samples was observed using a scanning electron microscope (Nova NanoSEM 230, FEI, Hillsboro, OR, USA). Samples were attached to the operating platform with double-sided conductive adhesive tape, then covered with a thin layer of gold. The morphology was determined at an accelerating voltage of 5 keV [31].

### 2.7. Pasting Properties

Pasting profiles were determined as described previously, with minor modifications [32], using a Rapid Visco Analyser (Anton Paar GmbH, Graz, Austria). Lotus seed starch–protein mixtures (2.0 g) were accurately weighed and suspended in deionized water (25 mL) to a final concentration of 8%. The sample was weighed on the dry basis of native LS in the aluminum RVA sample canister. A programmed heating and cooling cycle was used where the samples were pasted at an initial temperature of 50 °C for 1 min, heated to 95 °C at 5 °C/min, held for 10 min, cooled to 50 °C at 5 °C/min and held for 2 min.

### 2.8. Thermal Properties

The pasting thermal parameters of the samples were measured by DSC (DSC 214 Polyma, Netzsch, Selb, Germany). Distilled water (10 μL) was added to the sample (3 g), in a sealed crucible, which was left to stand at ambient temperature for 12 h. An empty crucible was used as the reference. To make a measurement, the temperature was raised from 25–150 °C, at 10 °C/min.

### 2.9. Swelling Power and Solubility of Mixtures

The emulsion (2%, LS-LP mixtures (1 g)) was added to distilled water and oscillated in a water bath at different temperatures (25, 65 and 95 °C) for 30 min. The dried aluminum boxes, which contained supernatant from the centrifuge test tubes (5000× *g*, 10 min), were dried to constant weight, and then the weight gain of the aluminum boxes was recorded as the mass of dissolved starch (*A*), the mass of precipitate in centrifuge tube was recorded as the mass of starch that had swelled (*P*). We calculated the swelling power (*B*) and solubility (*S*) according to the formulae:

(1) $$S\;\left(\%\right)=\frac{A}{W}\times 100$$

(2) $$B\;\left(\%\right)=\frac{P}{W\left(1-S\right)}\times 100$$

### 2.10. Data Analysis

All experiments were performed in triplicate. All pictures were obtained by Origin software (OriginPro 2018C, MicroCal, Northampton, MA, USA) transformation based on the experimental data. Part data were analyzed using one-way analysis of variance (ANOVA), which was performed with Statistical Product Service Solutions (SPSS) software (IBM SPSS Statistics 23.0, IBM, Armonk, NY, USA). Statistically homogeneous subsets were identified with a post-hoc Tukey HSD multiple comparison test at α = 0.05. Results are expressed as means ± standard deviations.

## 3. Results

### 3.1. FTIR Spectroscopy

**Figure 1:**
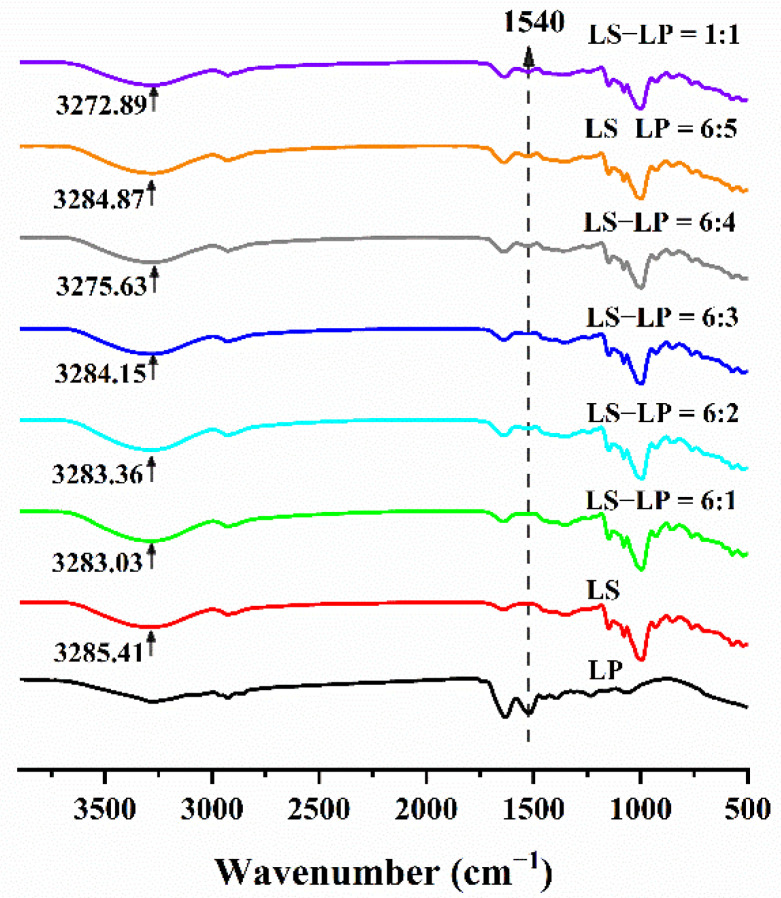
Infrared absorption spectra of lotus seed starch–protein mixture. Note: The figure displayed that lotus seed starch–protein mixture (LS-LP). LS indicates lotus seed starch. LP indicates lotus seed protein. LS-LP = 6:1–5 or 1:1 indicates lotus seed starch–protein with different ratios.

Infrared absorption spectra of LS), LP and LS-LP mixtures were recorded (Figure 1). The absorption peak at about 1540 cm^−1^, which was attributed to the amide II band of the imino group (N-H) of LP, was observed in all the spectra except that of LS (Figure 1); its intensity increased with increased protein addition, which indicates that added protein provides more N-H bonds for LS-LP interactions, but the strength is lower than in LP. The increased intensity of the amide II band was attributed to electrostatic interactions between the amino group of LS and the carboxyl group of LP, in agreement with a previous report [33]. The absorbance peak of LS hydroxyl groups (O-H) in the spectra of mixtures (Figure 1) showed a down-shift to the lower wavenumber range of 3000–3600 cm^−1^, compared with LS, which is attributed to the electrons in the hydrogen bond structure being delocalized, which can give a lower bond force constant, resulting in the stretching vibration of the hydroxyl group involved in the hydrogen bond being expected to have a lower wavenumber than the non-hydrogen-bond counterpart, thus reflecting the hydrogen bond density and strength [5]. Therefore, under the interaction of starch and protein, the molecular mobility decreases, indicating a significant increase in hydrogen bond density and strength.

As discussed above, the characteristic peaks of LS did not shift noticeably, indicating that the addition of LP did not change the starch structure, and no new peaks were produced by adding protein, indicating that there were no new covalent bonds between LS and LP. In general, the interactions between LS and LP appear to be predominantly through hydrogen bonds, and the number and strength of hydrogen bonds increase with increasing protein addition. However, the interactions between LS and LP may also include van der Waals forces and electrostatic interactions [18].

### 3.2. UV–Visible Absorption Spectra

**Figure 2:**
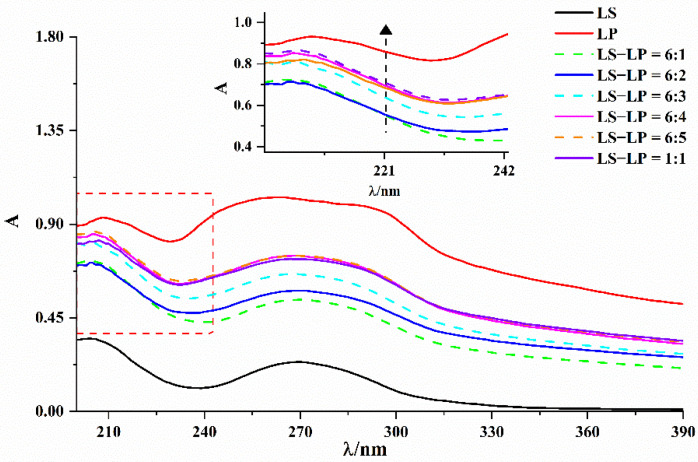
UV–vis absorption spectra of lotus seed starch–protein mixture. Note: The figure displayed that lotus seed starch–protein mixture (LS-LP). LS indicates lotus seed starch. LP indicates lotus seed protein. LS-LP = 6:1–5 or 1:1 indicates lotus seed starch–protein with different ratio.

UV–visible absorption measurement is a very effective method to explore protein structure and mixture formation [34]. The absorption band of protein secondary structure, at 200–240 nm reflects the interactions between protein and other substances [16]. The absorption spectra of lotus seed starch–protein mixtures (LS-LP, Figure 2), had one main absorption peak in the 200–240 nm range and the highest peak of this absorption band is about 208 nm. The LP absorption spectrum displays the highest intensity, and that of LS had the lowest intensity. The intensity of the main absorption peak positively correlated with the amount of protein added until an LS/LP ratio of 6:4 and the intensity was close to the maximum value, but the intensity was always lower than that of LP. It appears that LP could interact with the amylose in LS, which modifies the secondary structure of the protein, and the higher the concentration of protein, the stronger the interactions with amylose [1]. Similar changes were observed in the UV–visible absorption spectrum of the protein in soluble-starch–whey-protein isolate mixtures [16].

### 3.3. SEM

**Figure 3:**
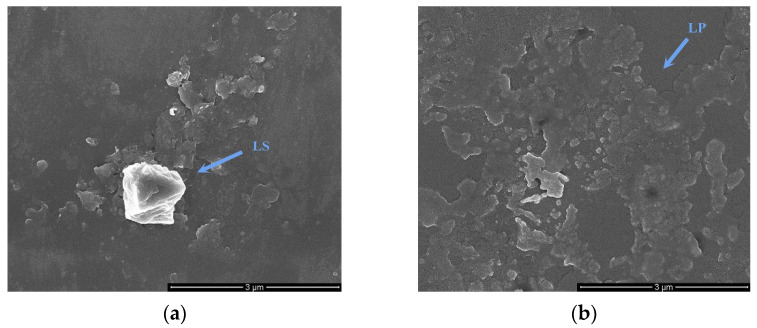
The scanning electron microscopy (SEM) image of lotus seed starch (**a**) and protein (**b**) (×50,000).

Lotus seed starch (LS) exists as starch granules in the plants, but the shells of the granules were disrupted after gelatinization and no intact granules were observed by SEM. Disruption of the granules results in dissolution and gelation of amylose and amylopectin, which can be linked by hydrogen bonds to form a crystalline structure on cooling [35], which is displayed in Figure 3a. In addition, the lotus seed protein (LP) denatures and aggregates during heating (Figure 3b).

**Figure 4:**
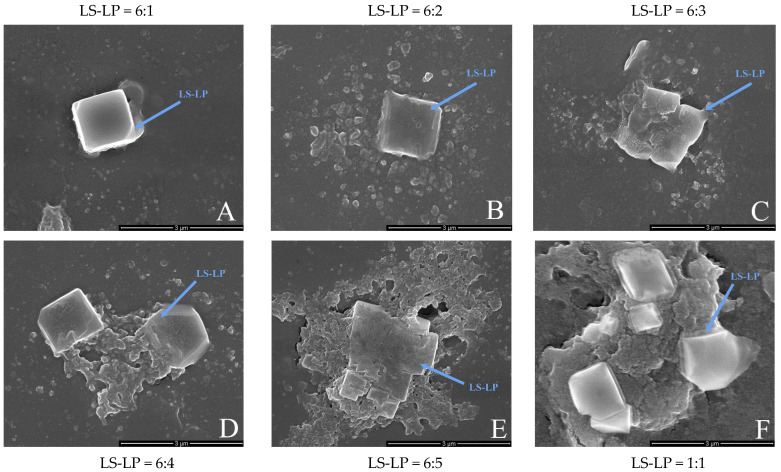
The scanning electron microscopy (SEM) image of lotus seed starch–protein mixture (×50,000). Note: Picture (**A**−**F**) indicates the different ratio of lotus seed starch–protein (LS-LP = 6:1–5 or 1:1).

The surface morphology of the LS-LP mixture was observed by SEM (×50,000, Figure 4). The apparent crystalline granular structure is mainly formed from starch crystals, and the amorphous, aggregated structures may be aggregates of the LS-LP mixture (Figure 4A–F). As the protein content of the mixtures increased, the relative abundance of crystals decreased and the crystals were progressively wrapped through the aggregates of the mixture. At an LS/LP ratio of 1:1, the individual crystals were almost completely encapsulated by aggregation and appeared very extensive. Similar results were reported previously [35]. In addition, it is clear that, as the protein content increases, more mixture aggregates encapsulate the crystals. At an LS/LP ratio of 6:4, larger-scale aggregation became apparent, mainly visible around the crystals. With further increases in protein content, aggregation increased further and the crystals were completely surrounded by aggregation. Most of the starch crystals were buried in the aggregates, with a few visible on the surface of the aggregates, but aggregation was also observed at the edge of the crystals. Ribotta et al. [36] found that with the increase in soybean protein concentration, wheat starch particles would be more tightly wrapped by soybean protein. Combined with the results of FTIR, indicate that starch may attract a small part of protein molecules to be mixed in the crystal through electrostatic interaction during forming crystal structure, while most of the remaining protein molecules will form aggregates through electrostatic interaction with starch molecules that did not participate in the formation of a crystal, which are distributed around the crystal and wrapped with the crystal, and the connection between starch and protein molecules may include a hydrogen bond [37]. Gui et al. [18] observed that potato protein was not uniformly attached to the surface of potato starch particles through electron microscopy. Taken together, these results indicated that the electrostatic interactions between LS and LP formed dispersions of starch-based microcrystals in protein-based aggregates.

### 3.4. XRD

**Figure 5:**
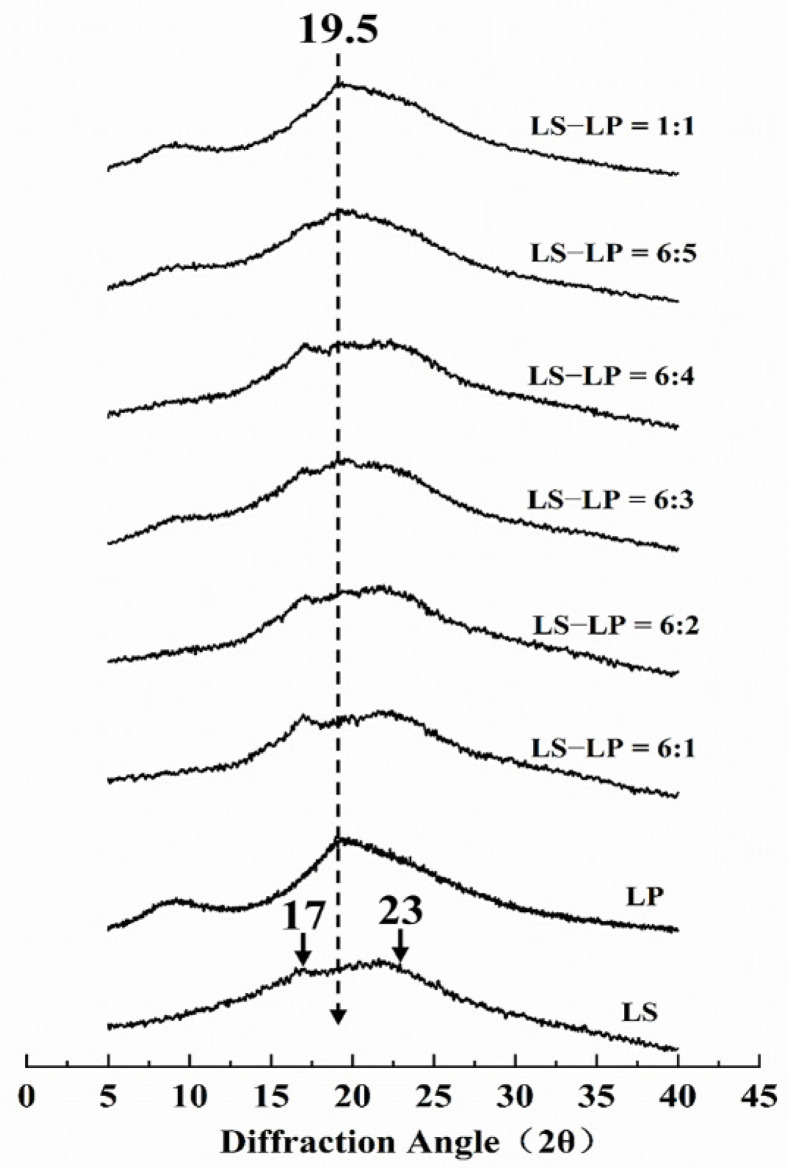
The X-ray diffraction pattern of lotus seed starch–protein mixture. Note: The figure displayed that lotus seed starch–protein mixture (LS-LP). LS indicates lotus seed starch. LP indicates lotus seed protein. LS-LP = 6:1–5 or 1:1 indicates lotus seed starch–protein with different ratios.

The information of starch granule crystalline can be classified into “A”, “B” and “C” types. Type A starch displays stronger diffraction peaks at around 2θ = 15°,17°,18° and 23°; the X-ray patterns of type B starch give the strongest diffraction peak at 2θ = 17°; and the type C starch is a mixture of both A-type and B-type patterns [38]. The XRD patterns of the LS-LP mixtures were recorded (Figure 5). LS showed a strong diffraction peak at 17° and 23° (2θ) which indicated that the gelatinized LS has a typical C-type crystal structure. Another strong diffraction peak appeared at 19.5° (2θ) after adding protein, which is linearly related to the LS/LP ratio. However, the original peaks at 17° and 23° (2θ) gradually disappear until the LS/LP ratio at 1:1. 

When the ratio of LS/LP at 6:1–2, the two obvious peaks (17°, 23°) were mild than that of gelatinized LS and no absorption peak of protein displayed in the XRD patterns, which indicates that starch is still dominant component in the LS-LP mixture system and the protein at a low concentration. 

The ratio of LS/LP at 6:3–4 displays the stronger absorption peak (19.5°) of protein and weaker absorption peaks (17°, 23°) of starch. It indicates that with the increase in protein concentration, the interaction between more protein and starch may lead to cross-linking, which could produce more crystalline blends and reduce the interaction between starch and starch. Therefore, the strength of starch absorption peaks (17°, 23°) becomes gradually weak [13]. Meanwhile, the appearance of protein absorption peak indicates that there exists good biocompatibility between two biomacromolecules [39].

In the XRD pattern of the blend with the ratio of LS/LP at 6:5 or 1:1, only the characteristic protein peak (19.5°) was observed, no obvious peaks characteristic of the starch were observed, which may be due to interaction between starch and more protein [39]. This interaction could change the hydrogen bond in the LS-LP mixture system and transform the formation of the crystal region [40]. On the other hand, the reason for this phenomenon can be explained that LP can encapsulate LS crystals, forming a protective film on the crystal surface; the higher the protein content, the thicker the film, which increasingly inhibits the interaction between LS and water, and strengthens the interactions between LS and LP, which is consistent with the results of SEM. In addition, the absorption peak intensity of the XRD pattern indicated the aging degree of starch after gelatinization. The shorter the diffraction peak, the weaker the aging degree [41]. Therefore, when the protein concentration reached a high level, it can inhibit the aging of starch, which is consistent with the results observed by RVA.

### 3.5. Pasting Properties

**Figure 6:**
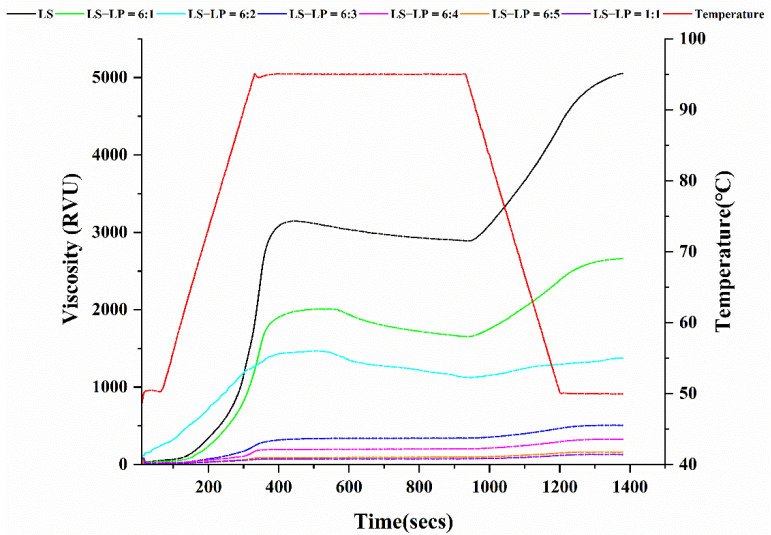
The pasting curve of lotus seed starch–protein mixture. Note: The figure displayed that lotus seed starch–protein mixture (LS-LP). LS indicates lotus seed starch. LS-LP = 6:1–5 or 1:1 indicates lotus seed starch–protein with different ratios.

The pasting properties of LS-LP mixtures were tested with a rapid visco-analyzer, to determine the pasting temperature, peak viscosity, hold-through, final viscosity, breakdown and setback (Table 1, Figure 6) [36]. The pasting curve is displayed in Figure 6. All of the pasting parameters, except for the pasting temperature, decreased as the LS/LP ratio increased. The decreased peak viscosity of LS-LP mixtures (Table 1) may result from the addition of protein, which inhibits the swelling and disintegration of starch particles by electrostatic interaction. A similar result was reported on the effect of casein and its hydrolysates on the peak viscosity of corn starch [3]. 

The breakdown viscosity is typically used as an indicator of the stability of gelatinized starch [42]. The breakdown of LS-LP mixtures initially increased as the protein content increased (Table 1), indicating that the protein molecules compete with starch molecules for water molecules, thereby influencing the structure and stability of the mixture. The reduction in setback by further increases in protein content appears to be related to the spatial network structure formed between protein molecules and to the encapsulation of the starch crystals by protein; both of these interactions appear to improve the stability of the LS-LP mixture, thereby improving its thermal stability, until the system reaches saturation, when LS/LP = 6:3. These results are consistent with a previous report [43] and with the SEM observations. Setback is related to the degree of recrystallization of the starch after gelatinization, which is an indicator of its storage stability [44]. The reduction in setback had a linear relationship with protein content, indicating that the gelation and reorganization behavior of starch molecules is hindered by the presence of protein, thereby inhibiting starch retrogradation and improving its storage stability.

### 3.6. DSC

**Figure 7:**
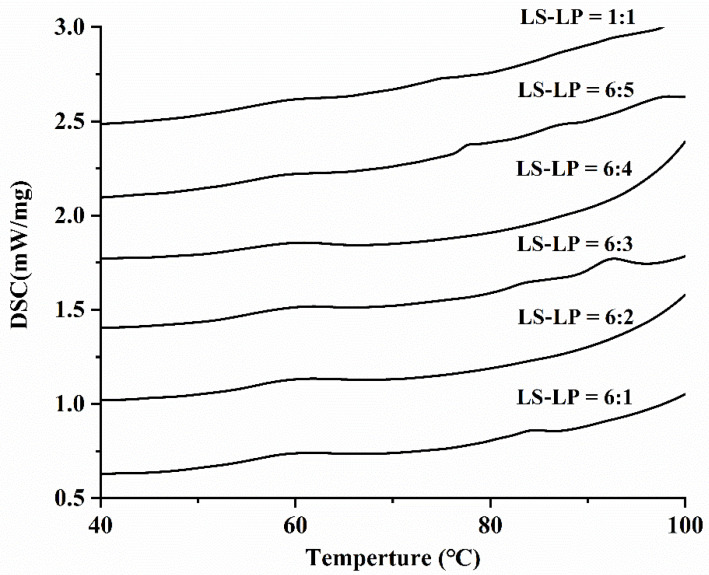
The pasting curve of lotus seed starch–protein mixture. Note: The figure displayed that lotus seed starch–protein mixture (LS-LP). LS-LP = 6:1–5 or 1:1 indicates lotus seed starch–protein with different ratios.

The thermal properties of LS-LP mixtures were analyzed by DSC and the enthalpy change (ΔH) and gelatinization temperatures, i.e., the onset temperature (To), peak temperature (Tp), and cease temperature (Tc) were determined (Table 2, Figure 7). The To and Tp increased with increased protein content, whereas the Tc and ΔH decreased. Interactions between LP and LS molecules resulted in LP competing with LS for water, inhibiting hydration of starch molecules and consequently increasing To and Tp. The reduction in Tc and ΔH of LS-LP mixtures with increasing protein content takes place in three stages. Initially, when little protein is present (LS/LP = 6:1 or 6:2), there are plenty of water molecules to hydrate the starch, resulting in a slight decrease in Tc and ΔH. With the further addition of protein (LS/LP = 6:2 or 6:3), most water molecules are absorbed by the protein and there is insufficient water to hydrate the starch molecules, decreasing Tc and ΔH significantly. When LS/LP increases to 6:4 or 1:1, the abundant protein can form a stable network structure, reducing the ΔH, but keeping Tc relatively stable. This indicates that the interaction between LP and water molecules reaches saturation and there is no additional competition with starch molecules. Overall, the addition of protein hinders the gelatinization of starch, because the competition for water molecules between starch and protein leads to the redistribution of water [5]. In addition, increased protein concentration results in the formation of an extensive network structure between protein molecules, which interferes with starch hydration, changes its thermal properties and significantly decreases its ΔH [14].

### 3.7. Solubility and Swelling Degree

**Figure 8:**
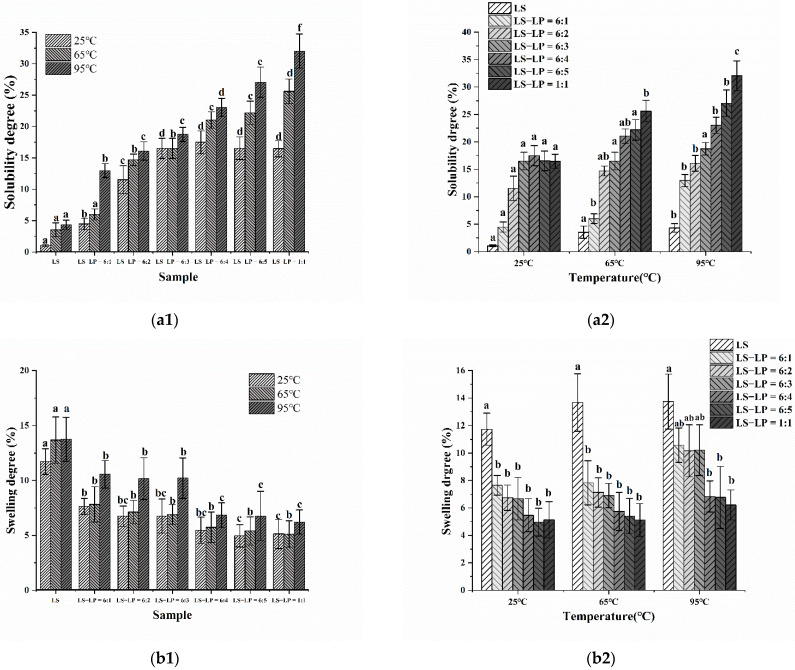
The solubility (a) and swelling (b) degree of lotus seed starch–protein mixture at different temperatures (**a1**,**b1**) (25 °C, 65 °C, 95 °C) and different samples at the same temperature (**a2**,**b2**). Note: LS indicates lotus seed starch. LS-LP = 6:1–5 or 1:1 indicates lotus seed starch–protein with different ratios. Mean values with different superscript in a same temperature (a1,b1) and same ratio (**a2**,**b2**) are significantly (p ≤ 0.05) different from each other.

The solubility and swelling degree of starch reflect the strength of interaction between starch and water molecules and the inter-chain interaction strength between starch chains in the amorphous and crystalline regions [45]. Changes in the solubility and swelling degree of LS-LP mixtures were determined as a function of temperature during heating at 25, 65 and 95 °C, which represent room temperature, the gelatinization temperature and fully gelatinized, respectively (Figure 8a1–b2). The solubility and swelling degree of LS-LP mixtures with different LS/LP ratios increased with increasing temperature. However, at a given temperature, the swelling degree of LS-LP mixtures decreased and the solubility increased, as the LS/LP ratio increased. It appears that the decreased swelling results from strong interactions between starch and protein, so the protein encapsulates the starch particles, inhibiting the entry of water and the leaching of amylose [46]. This result is consistent with the SEM results. In addition, strong hydrogen bonding between starch and other molecules contributes to the reduced swelling [47], consistent with the FTIR results. The protein molecules involved in encapsulating starch particles are more extensively hydrated and dissolve in water, which increases their solubility.

## 4. Conclusions

Starch and protein are the main nutritional components of lotus seeds. LS-LP interactions during heat treatment modified the starch structure at the molecular and particle scales, as well as its thermal and gelatinization properties. Electrostatic interactions and hydrogen bonding appear to be the main bonding interactions between starch and protein. With increasing protein content in the LS-LP mixture, the interactions between crystalline starch granules and protein aggregates strengthened. It appears that starch crystals attract proteins through electrostatic interactions, then the protein adsorbs onto the starch surface, where it binds by hydrogen bonding, resulting in the protein encapsulating the starch crystals. Thermodynamic analyses indicated that the swelling and gelatinization of the starch granules were inhibited by competition for water by the protein, which improved the pasting properties and may increase the potential for industrial applications of lotus seed. These findings provide a practicable way to control the thermal and gelatinization properties of LS-LP mixtures, by changing the proportions of the two components, and provide a theoretical basis for developing novel and functional lotus seed-based foods.

## Figures and Tables

**Table 1:** Pasting properties of lotus seed starch–protein mixture.

Samples	Peak Viscosity	Hold-Through	Breakdown	Final Viscosity	Setback
LS	3146	2902	244	5053	2151
LS:LP = 6:1	2010	1666	344	2663	997
LS:LP = 6:2	1467	1126	341	1374	248
LS:LP = 6:3	342	339	3	506	167
LS:LP = 6:4	203	197	6	325	128
LS:LP = 6:5	96	92	4	160	68
LS:LP = 1:1	75	72	3	128	56

(Note: The figure displayed that lotus seed starch–protein mixture (LS-LP). LS indicates lotus seed starch. LS-LP = 6:1–5 or 1:1 indicates lotus seed starch–protein with different ratios.).

**Table 2:** Thermal properties of lotus seed starch–protein mixture.

Samples	To(°C)	Tp(°C)	Tc(°C)	ΔH(J/g)
LS:LP = 6:1	50.8	68.2	58.9	2.582
LS:LP = 6:2	51	68.0	59	2.525
LS:LP = 6:3	51.1	65.7	59.2	1.946
LS:LP = 6:4	51.2	65.8	59.3	1.847
LS:LP = 6:5	51.4	65.9	59.4	0.9878
LS:LP = 1:1	51.7	65.9	59.6	0.8843

(Note: The figure displayed that lotus seed starch–protein mixture (LS-LP). LS-LP = 6:1–5 or 1:1 indicates lotus seed starch–protein with different ratios. To: onset temperature; Tp: peak temperature; Tc: cease temperature; ΔH: gelatinization enthalpy.).

## Data Availability

Data sharing is not applicable to this article.
